# Olfactory brain activations in patients with Major Depressive Disorder

**DOI:** 10.1038/s41598-023-36783-0

**Published:** 2023-06-21

**Authors:** Theresa Herrmann, Carina Koeppel, Jennifer Linn, Ilona Croy, Thomas Hummel

**Affiliations:** 1 Department of Otorhinolaryngology, Smell and Taste Clinic, TU Dresden, Dresden, Germany; 2 Department of Clinical Psychology, Friedrich Schiller University, Jena, Germany; 3 Department of Neuroradiology, TU Dresden, Dresden, Germany

**Keywords:** Medical research, Psychology

## Abstract

Depression is associated with reduced olfactory function. This relationship is assumed to be based on either a reduced olfactory bulb volume or diminished functioning of higher cortical areas. As previous results are controversial, we aimed to re-evaluate central olfactory processing in depression. We recorded the BOLD signal of 21 patients with Major Depressive Disorder and 21 age and gender matched healthy controls during odor presentation. In addition, we measured the individual olfactory bulb volume, tested odor identification and odor threshold, and asked for hedonic odor perception. In both groups, odor presentation led to a pronounced activation of primary olfactory areas. However, secondary olfactory areas were significantly less activated in depressed individuals. The two groups did not differ in olfactory bulb volume. Our results point towards altered olfactory processing in patients in those regions that relate to sensory integration and attention allocation. Difficulties in cognitive processing could impact olfactory function in depression. We are therefore in favor of a top-down mechanism originating in higher cortical areas explaining parts of the relation between depression and olfaction.

## Introduction

Depression is a serious medical condition that accounts for considerable disability. The burden of Major Depressive Disorder (MDD) is increasing worldwide^1,2^. Consequently, research on the neural mechanisms that potentially trigger or sustain the disorder has become increasingly important. This also includes studies on the association between depression and olfactory dysfunction.

In humans, abnormalities of olfactory function accompanying depressive behavior have frequently been investigated using psychophysical olfactory tests. As a range of methods has been employed, definitive conclusions cannot be drawn easily. Considering the various domains of olfactory perception, in depressed patients reduced olfactory threshold, identification and/or discrimination as well as altered hedonic perception of odors has been reported by several studies. Others however demonstrated no or very small differences in olfactory function (for a detailed overview see Ref.^3^). Deficits in odor identification and discrimination could be attributed more to cognitive processes, whilst altered olfactory threshold suggests more of an impairment at the level of the olfactory epithelium^4^. Although there is no consistency as to which of the olfactory domains is affected, recent reviews overall agree that depression is typically accompanied by at least partially compromised olfactory ability^3,5,6^.

Potential links of the olfactory system and depression on an anatomical level are appealing. A close relationship between olfaction and emotion processing can generally be expected in humans, as central olfactory and emotional processing pathways share mutual brain regions, such as amygdala, hippocampus, insula and orbitofrontal cortex^7^. While this overlap of relevant brain areas has often been suggested to be a potential driver for alterations found in olfactory tests in depression^8^, it is in fact only sparsely examined using neuroimaging. As several studies observed alterations in olfactory ability, it is intriguing to investigate the extent to which depression may change the processing and perception of olfactory information at the neuronal level. For this purpose, the classification into primary (receiving input directly from the olfactory bulb [OB]: piriform cortex, entorhinal cortex, amygdala) and secondary (orbitofrontal cortex [OFC], insula, thalamus, hippocampus) olfactory areas (as defined by Ref.^9^) proved suitable. Keeping in mind that depression is associated with abnormal activations in different brain areas such as the orbitofrontal cortex, the prefrontal cortex and the amygdala, and that these are also involved in olfactory perception, one could reasonably expect their dysfunction in depressive disorders to have an impact on olfactory function^10^. To date there is just one study investigating the association between depression and olfaction using MRI. Here, depressed women exhibited reduced activation in higher order processing areas^11^. Besides thalamus and insula, activation within the orbitofrontal cortex was found to be decreased in patients. However, it has to be noted that this study had some limitations. One of those is the selective sample of exclusively female depressed patients, another one is the—from today’s perspective—rather poor imaging resolution in the 1.5Tesla scanner and the application of uncorrected statistical thresholds, which are prone to false positive results. Considering that, the neural processing of odors in patients with depression is still not well understood and requires further imaging evidence.

The interplay of depression and olfaction can eventually be approached from different angles. First, one could argue that their relationship might be mediated by higher cognitive effects implying a top-down-mechanism. Electrophysiological tests with olfactory stimuli have indicated a reduction of endogenous processes including subjective emotional evaluation and attention in depressed patients^11^. It has been shown that olfactory perception can be affected by inducing a negative emotional state^12,13^. The negatively biased associative cognition in patients with MDD appears to affect mental processes such as attention^14^. It prompted the idea that this bias could likewise have an impact on sensory functions including olfaction through neuroanatomical projections^6^. The OFC, as an area associated with cognition, higher order olfactory processing as well as depressive processes^10^, seems most promising as neural correlate. As the central neocortical target of the olfactory cortex, the OFC receives direct afferents from all primary olfactory areas with projections bypassing the thalamic filter^9^. The OFC facilitates complex information processing including multimodal sensory integration and value attribution to olfactory stimuli^15^, allocation of attentional resources^16^, olfactory awareness and conscious perception^17^ and evaluative odor judgements^18^. With respect to the aforementioned preceding study^11^, a reduced activation of the OFC in depressed patients can also be expected for olfactory stimulation. Reduced metabolic activity in frontal cortical regions is frequently reported in studies investigating depression and believed to lead to secondary impairments in subcortical areas^19^. The acknowledgment of a so-called hypofrontality has even led to new therapeutic approaches for drug-refractory depression such as transcranial magnetic stimulation (TMS). On the assumption that targeted stimulation of the frontal cortex has a regulatory downstream effect on the limbic circuit, studies demonstrate robust clinical improvement of depressive symptoms^20^. Interestingly, TMS has also been reported to improve olfactory function in MDD patients^21^. Abnormalities in frontal cortex activation are reported to decrease over the course of therapy^22^. A corresponding observation is that changes in olfactory abilities improve with antidepressant therapy^11,23,24^, possibly related to augmented utilization of cognitive and attentional resources. As such, observed deficits in olfactory function in depressed patients could be considered a state rather than a trait marker^3^.

Second, one might suggest that the olfactory system modulates emotional responses via the connections of the OB to the limbic circuit. Animal studies showed that experimental removal of the bilateral olfactory bulb (OB) leads to depression-like behavior^25^. Previous research from our group indicated that patients with MDD have a relatively small OB^26,27^. The reduced olfactory input to limbic circuits in individuals with a small OB volume has been proposed to result in the need for a stronger emotional stimulus to trigger supra-threshold action potentials, which may explain an increased vulnerability to depression^3^. Supporting this hypothesis, people who lost their sense of smell show weaker arousal and reduced limbic processing to visually presented emotional stimuli^28^. It has been speculated that, if the OB is rendering people susceptible to depression, a reduced OB volume would result in reduced signaling to subsequent olfactory areas, which in turn would disrupt healthy emotional functioning^3^. If so, a reduced BOLD signal in downstream brain areas could be expected for olfactory stimulation.

Aim of this study was to investigate MDD patients’ BOLD response in primary and secondary olfactory areas to olfactory stimulation in view of our considerations presented above. A reduced olfactory evoked BOLD response in olfactory areas would be expected for the MDD patient group compared to healthy controls. Psychophysical and olfactory tests and odor ratings are used for supportive assessment. Regarding the two previously outlined approaches concerning the relationship between olfaction and depression, two alternative scenarios are imaginable. Altered activation in secondary olfactory areas and brain regions responsible for higher cognitive tasks would rather support the first approach, whereas altered activation of primary olfactory areas directly connected to the OB would mainly support the second approach.

1. Top-down-approach: We assume a reduced activation of mainly higher order olfactory areas. With the background of hypofrontality in depressed patients, frontal olfactory areas seem to be particularly promising. Difficulties in cognitive processing could impact sensory functions in depression.
2. Bottom-up-approach: We hypothesize that reduced volume in the OB is related to increased vulnerability to depression and to reduced neuronal processing in olfactory areas. We therefore test in a sample of MDD patients whether those patients have I) a reduced OB volume compared to age- and sex-matched controls and II) whether the neural response in downstream brain areas is related to OB volume.

## Materials and methods

### Participants

Twenty-one patients (twelve women; all right-handed as to the patients’ self-assessments; aged 18 to 62 years; M = 39.2 years; SD 12.0) with a history of MDD (ICD10: F32/F33) were included in this study. Two patients were diagnosed with a depressive episode (F32), nineteen patients with a recurrent depressive disorder (F33). Further mental and behavioral disorders prevalent in patients include phobic anxiety disorder (n = 9), obsessive compulsory disorder (n = 4), somatoform disorder (n = 1), generalized anxiety disorder (n = 1) and dysthymic disorder (n = 3). Before participating, a detailed standardized Structured Clinical Interview for DSM-IV Axis I Disorders SCID-I^29^ was performed by trained interviewers. At the time of investigation, nine patients did not use antidepressant medication, whilst twelve received antidepressant drugs. For a detailed description of patients’ characteristics, please refer to Table 1.

**Table 1:** Characteristics of participants.

	Healthy controls (n = 21)		MDD patients (n = 21)		d	p
	Mean	SD	Mean	SD		
Age (years)	38.8	12.1	39.2	12.0	− 0.03	0.98
BDI-II depression score	2.7	2.5	29.6	9.2	− 4.00	< 0.001
SHAPS pleasure scale	1.1	2.4	5.4	3.2	− 1.52	< 0.001
Odor threshold	10.4	2.5	9.0	3.2	0.48	0.13
Odor identification	13.8	1.8	13.7	1.2	0.06	0.84
Odor discrimination	9.0	0.0	8.6	0.6	0.68	0.002
OB volume	45.0	12.7	50.3	12.5	− 0.42	0.182

	Mean	SD	Mean	SD	r	p
Odor discrimination	9.0	0.0	8.6	0.6	0.68	0.002

Patients showed reduced scores for olfactory threshold and identification. However, this difference could not be proven as significant. Patients performed significantly worse in the odor discrimination task. No significant group difference was observed for the OB volume.

*MDD* patients diagnosed with Major Depressive Disorder, *BDI-II* Beck Depression Inventory-II; *SHAPS* = Snaith-Hamilton Pleasure Scale, *SD* standard deviation; p as tested with the t-test for independent samples respectively the Mann–Whitney U test; *d* Cohen’s d, *r* rank-biserial correlation.

A group of twenty-one healthy participants (twelve women; all right-handed; aged 18 to 60 years; M = 38.8 years; SD 12.1) carefully matched for age and gender served as controls. The control group was recruited via our participant database and public announcements. We enrolled interested participants on a “first-come, first-served” basis, who met the inclusion criteria (absence of depression) and matched in age and sex to one of the participants in the patients group.

Absence or presence of depression served as inclusion criteria (patients: BDI questionnaire ≥ 13 + diagnosis of major depression; controls: BDI questionnaire score < 13). Exclusion criteria for all participants were neurological disorders, history of craniocerebral trauma, nasal disorders (e.g. cold, allergies, sinonasal surgery), smoking, pregnancy and eating disorders. Additionally, healthy volunteers were required to have no current or previous psychiatric diseases.

On the day of MRI, depression severity was assessed using the German version of the Beck’s Depression Inventory BDI II^30,31^ for both patients and healthy participants and all participants completed the German Version of the Snaith Hamilton Pleasure Scale SHAPS-D^32,33^ focusing on anhedonia symptomatology. As expected, patients presented high BDI and SHAPS scores compared to the control group (BDI t(40) = 12.96, p < 0.001); SHAPS t(40) = 4.92, p < 0.001). Depression scores also explained a significant proportion of variance in anhedonia as measured with the SHAPS-D (R^2^ = 0.51, F(1,124) = 129.38, p < 0.001).

### Assessment of olfactory function

All participants were tested for olfactory function using the “Sniffin’ Sticks” test battery (Burghart GmbH, Holms, Germany; Ref.^34^) applying the subtests for olfactory identification and threshold. Odors are presented in pen-like dispensers. Odor identification ability was tested for 16 familiar odors. Participants were asked to identify each odor by selecting the best fitting descriptor from a list of four options, presented as text and picture. Each correctly identified odor scores one point, thus the maximum score is 16.

Birhinal olfactory threshold was assessed in a forced-choice paradigm presenting gradually higher concentrations of phenylethyl alcohol (PEA). First, participants were familiarized with the odor of PEA. Next, triplets of pens with two of them containing only solvent and one containing the target fragrance PEA were presented in a randomized fashion. Olfactory threshold was assessed in ascending order, starting from the lowest concentration of PEA. Once the odor-containing pen was correctly identified in two successive attempts, pens with lower PEA concentrations were offered. If the odor was not correctly identified, presented concentrations were reversed until correct detection was possible again. Threshold scores were defined as the mean of the last four reversals. The attainable score could range from 1 to 16. A high value implies a low olfactory threshold and hence better olfactory sensitivity^35^. Participants were blindfolded during the investigation and were not given any feedback on the accuracy of their answers until after the examination.

### Odor samples and olfactory stimulation during MRI session

A total of three odors was chosen: a typically unpleasant smell (tar: Fragrance Resources, Hamburg, Germany), a typically pleasant smell (lilac: Frey + Lau, Henstedt-Ulzburg, Germany) and a chocolate odor (Fragrance Resources, Hamburg, Germany), as food odors were shown to activate dopaminergic pathways in healthy individuals^36^. For all stimuli, clean air served as a baseline. Pleasantness of the odors was tested in a pilot study involving 11 subjects. Lilac and chocolate had consistently been rated as pleasant, whilst tar was perceived as unpleasant. Based on results from the pilot study (compare Supplementary Information), lilac and chocolate were kept at neat concentration, whilst tar was diluted to a 10% concentration using propylene glycol (Merck group, Darmstadt, Germany, product number 25265-71-8). This insured an isointense presentation of all odors during the experiment.

Subjects were familiarized with each of the odors prior to the scanning sessions and the odor rating scales for intensity and pleasantness were briefly explained.

During the fMRI recordings odors and clean air were delivered through a birhinal nasal cannula (constant airflow rate 2.5 l/min) to both nostrils independent of inspiration, using a computer-controlled, custom-built 3-channel olfactometer^37^. The odors were presented in counterbalanced order to the subject during a total of three runs, lasting 4 min each. Having chosen a conventional block design paradigm, each run was composed of 12 alternating ON and OFF periods. Within each run all three odors were presented in a randomized fashion, with a total of four blocks per odor. During ON-Blocks olfactory stimulation was switched on and an odor was delivered to the participant for 8 s, followed by the OFF Block with clean, odorless air being presented for 12 s to minimize olfactory adaptation (odor stimulation paradigm as described in Ref.^38^).The order of runs for odor presentation was additionally pseudorandomized across subjects.

**Figure 1:**
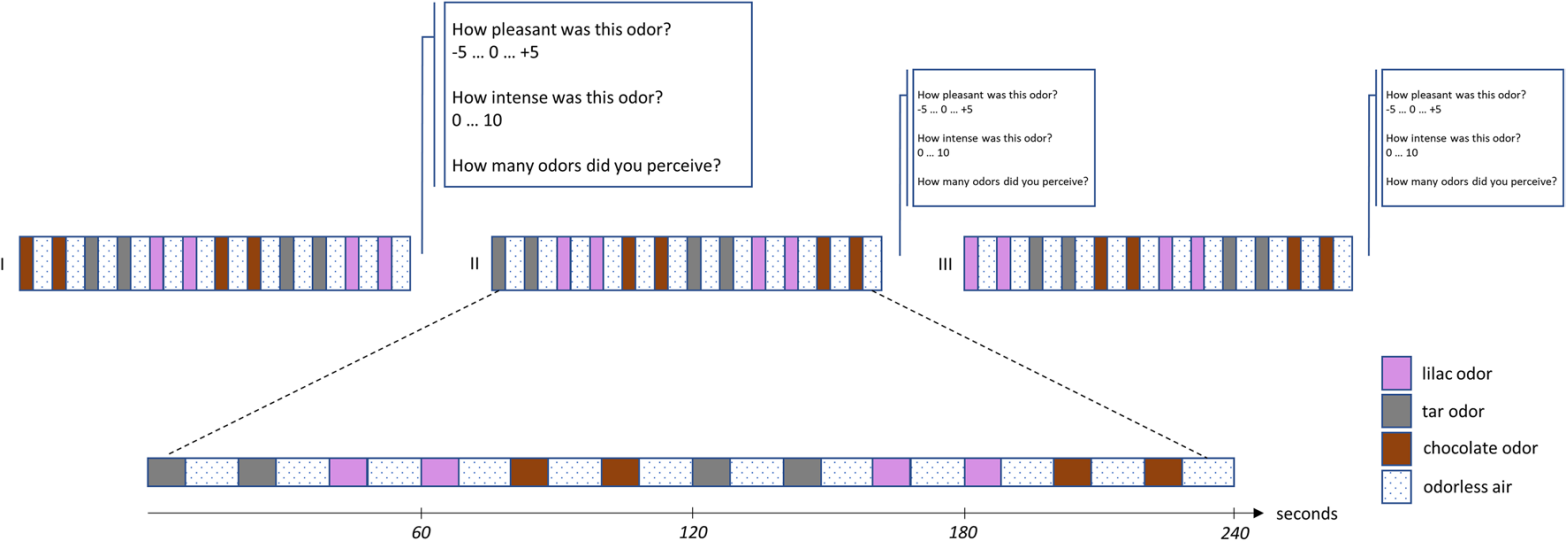
Odor Stimulation Paradigm. Three runs of 4 min each with a total of 4 presentations per odor per run were measured. Each run was followed by verbal ratings of the odors regarding intensity and pleasantness on a scale ranging from 0 (very weak) to 10 (very intense) or -5 (unpleasant) to 5 (very pleasant) respectively. Odors were presented in a block-design with alternating ‘olfactory stimulation’- and ‘clean odorless air’ periods. The run order was pseudorandomized across subjects.

During the fMRI procedure, the subjects were asked to stay calm and focus on the presented odors. Following each run the participants were asked to verbally rate each odor regarding intensity and pleasantness, with intensity scores ranging from 0 (not perceived) to 10 (extremely intense) and pleasantness scores ranging from -5 (extremely unpleasant) to 5 (extremely pleasant), respectively. (Fig. 1). Participants were also additionally asked to state the number of odors they had been able to clearly distinguish (0–3). The number of clearly perceived odors during the MRI scans according to individuals’ ratings after each scan was added up. Thus, the maximum attainable value when detecting all three odors in all three scan sessions is nine.

### Imaging procedure

Examinations were performed on a 3 T Siemens Magnetom Prisma Scanner (Siemens Healthcare, Erlangen, Germany) using a 32-channel head coil. Functional brain imaging was used to measure the blood-oxygenation-level-dependent (BOLD) signal after odor presentation as an indicator of brain activation. Functional images were acquired in an interleaved mode with a total of 24 slices per individual and the following parameters: TR = 1510 ms/TE = 30.0 ms; FoV = 220 mm; ISO voxel size = 2.5 × 2.5 × 2.5 mm; Flip angle 90°.

Following the fMRI sessions high resolution T1-weighted images were obtained using a three-dimensional, magnetization-prepared rapid gradient-echo (MP RAGE) sequence. A total of 160 slices was acquired with the following parameters: TR = 2300 ms/TE = 3.43 ms; FoV = 256 mm; TI = 900 ms; ISO voxel size = 1.0 × 1.0 × 1.0 mm; Flip angle 9°.

A complementary special T2-weighted sequence (QISS) was conducted for the measurement of the OB. The sequence was covering the anterior and middle segments of the skull with TR = 1000 ms/TE = 127 ms; FoV = 160 mm; ISO voxel size 0.5 × 0.5 × 0.5 mm; Flip angle 100°; no interslice gap.

### OB volume

Volumes of the left and right OB were obtained by manually contouring the size in every coronal plane using AMIRA 3D visualization and modelling system (Visage Imaging, Carlsbad, USA). Measurements were conducted blinded to the group category following a standardized protocol^39^. First, we selected those slices in which the OBs were visible in the coronal plane. The first anterior layer with a visually identifiable OB was used as starting point for the measurements. As commonly approved distal limitation we used the sudden change of diameter at the transition to the olfactory tract^40^. The outlines of the OBs were drawn manually in each successive slice. Volumes of both OBs were obtained in mm^3^ by multiplying the calculated OB surface of each slice with the slice thickness. All manual segmentations were performed by one observer. For reliability purposes, measurements were repeated by a second independent observer for half of the OBs (n = 21) to check for the inter-rater correlation (IRC = 0.83, p < 0.001).

As suggested in previous research, overall olfactory functioning seems to be mostly determined by the best nostril^41,42^ which is correlated with a higher corresponding OB volume^43^. Individuals’ highest (best) OB volume was therefore used for further analysis.

### Statistical analysis

Statistical data analyses were performed with SPSS 25 (SPSS Inc., Chicago, Ill., USA). Independent samples t-tests between controls and patients were performed for psychophysical data, odor ratings and number of correctly identified odors during the MRI runs as well as questionnaire results and OB volumes with level of significance set to 0.05. To account for violations of the normality assumption, differences in odor discrimination performance between healthy controls and MDD patients were calculated with the non-parametrical Mann–Whitney-U-Test. Hedonic ratings were further converted into absolute values, thereby negative and positive ratings would not cancel each other out. Repeated measurement ANOVA with two groups (controls vs patients) covering all three odors was performed for the resulting values. Effect size estimates are stated as Cohen’s d for the t-tests and as η^2^ for the F-test.

In order to estimate the impact of depression severity, Pearson correlations were calculated between the BDI and the odor threshold, identification ability, OB volume, odor intensity and the mean hedonic responses across all odors. Further, correlations of the OB volume and threshold ability, identification performance and the BOLD responses in primary and secondary olfactory regions of interest (ROI) were calculated.

### fMRI analysis

Data analysis was performed with SPM 12 software (Statistical Parametric Mapping, Wellcome Department of Imaging Neuroscience, London, UK), implemented in Matlab R2018a (Math Works Inc., Natick, MA, USA), following spatial pre-processing with the same software (realignment, co-registration of functional and structural images, normalization, smoothing by means of 8 × 8 × 8 mm^3^ FWHM Gaussian kernel). Based on the general linear modeling approach, SPM-matrices revealing the ON–OFF-differences were generated for each odor and participant. We used the final five seconds of odor stimulation for the "ON" condition and the final eight seconds of odorless air for the "OFF" condition, as similarly described in previous studies^38,44^. Doing so allows us to account for the possibility of a delayed arrival of the odors at participants' nostrils due to the tube length, but also to widely avoid a distorted activation due to a potential excess of odors into the odorless air baseline condition and vice versa. Motion parameters were included as covariates. Individual SPM-contrasts were incorporated into a full-factorial second level analysis, comprising the conditions “group” (patients, controls) and “odor” (lilac, tar and chocolate).

Given our research focus, the first step was to perform a ROI analysis for primary (receiving input directly from the OB; piriform cortex, entorhinal cortex, amygdala) and secondary (orbito-frontal cortex, insula, hippocampus, thalamus) olfactory areas (as defined by Ref.^9^). Respective masks were created using the probability tissue labels provided by Neuromorphometrics, Inc (Sommerville, Mass., USA). Joint activation across all three odors vs. baseline was assessed, separately for patients and controls, subsequently in contrast. Using the same approach, we performed a whole brain analysis to identify potential additional areas of relevant activation. Joint brain activation following odor exposure was assessed with a statistical threshold of p < 0.05 Family Wise Error (FWE) corrected. In line with previous studies^11^, the global height threshold was lowered to p < 0.001 (extent threshold k = 5) for the comparison between both groups. This liberal threshold was used in order to minimize the risk for false negative results in this first study of its kind. However, one has to keep in mind, that this approach simultaneously enhances the risk of false positive results. Activation coordinates are presented in MNI space.

The mean BOLD activation per ROI and odor condition in the FWE corrected model was further extracted for each individual using marsbar^45^. The data was used in a generalized linear mixed model (GLM) implemented in SPSS with the mean BOLD signal as target of the analysis, in which each subject served as an individual, and each ROI (n = 7) and odor (n = 3) served as a repeated measure. To analyze the differences between depressed and non-depressed subjects ROI were categorized into classes of primary and secondary olfactory areas. Residual method was used with model-based covariances. We modelled the main effect of group and the group by ROI class interaction effect. Post hoc tests were performed if the interaction effect reached an alpha level of < 0.1. Post hoc tests were performed as pairwise contrast (contrast type group) within the model, as embedded in SPSS 25.

### Ethics statement

The study was performed in accordance to the Declaration of Helsinki on Biomedical Research Involving Human Subjects and was approved by the Ethics Committee at the Medical Faculty of the Technical University Dresden *(EK 404102017)*. All participants provided informed written consent before taking part in the experiment. Control subjects received moderate payment for their participation.

## Results

### Psychophysical testing

On average, patients exhibited a reduced odor threshold score, however this missed significance (t(40) = 1.56, p = 0.13, d = 0.49)*.* Both groups showed a similar odor identification ability (t(40) = 0.205, p = 0.84). Results are reported in Table 1. Threshold scores and BDI showed a medium strong negative correlation that marginally missed significance (r = − 0.30, p = 0.052). No significant correlation of odor identification ability with the BDI was observed.

### Odor ratings in the context of MRI

Patients rated odors more neutral (0) as compared to controls (F(2,40) = 4.14, p = 0.019, partial η^2^ = 0.094). In contrast to hedonic ratings, intensity of all odors was similar for both groups (F(2,40) = 1.30, p = 0.28, partial η^2^ = 0.031). Hedonic ratings of the odors showed a significant negative correlation with the BDI (r = − 0.27, p = 0.002). Odor intensity ratings showed no significant correlation with the BDI.

In each of the three odor presentation runs, all three odors were presented and the number recognized by the participant was noted and subsequently totaled. Whereas all healthy participants were able to distinguish between the different odors during MRI sessions, eight patients exhibited difficulties discriminating between odor samples. There was a statistically significant difference in odor discrimination performance between healthy controls (M_Rank_ = 25.50) and MDD patients (M_Rank_ = 17.50), U = 136.5, Z = − 3.098, p = 0.002, r = 0.68.

### OB volume

OB volumes were not significantly different between healthy controls (45.0 ± 12.7mm^3^) and patients (50.3 ± 12.5mm^3^; t(40) = 1.36, p = 0.18, d = − 0.42). No significant correlations were found between OB volume and threshold ability (r = 0.05, p > 0.05), identification performance (r = 0.01, p > 0.05), BDI (r = 0.24, p > 0.05) and SHAPS (r = 0.20, p > 0.05). No significant correlations of the OB volume with extracted BOLD signals in any of the downstream primary or secondary olfactory areas were observed.

### Olfactory functional magnetic resonance imaging

**Figure 2:**
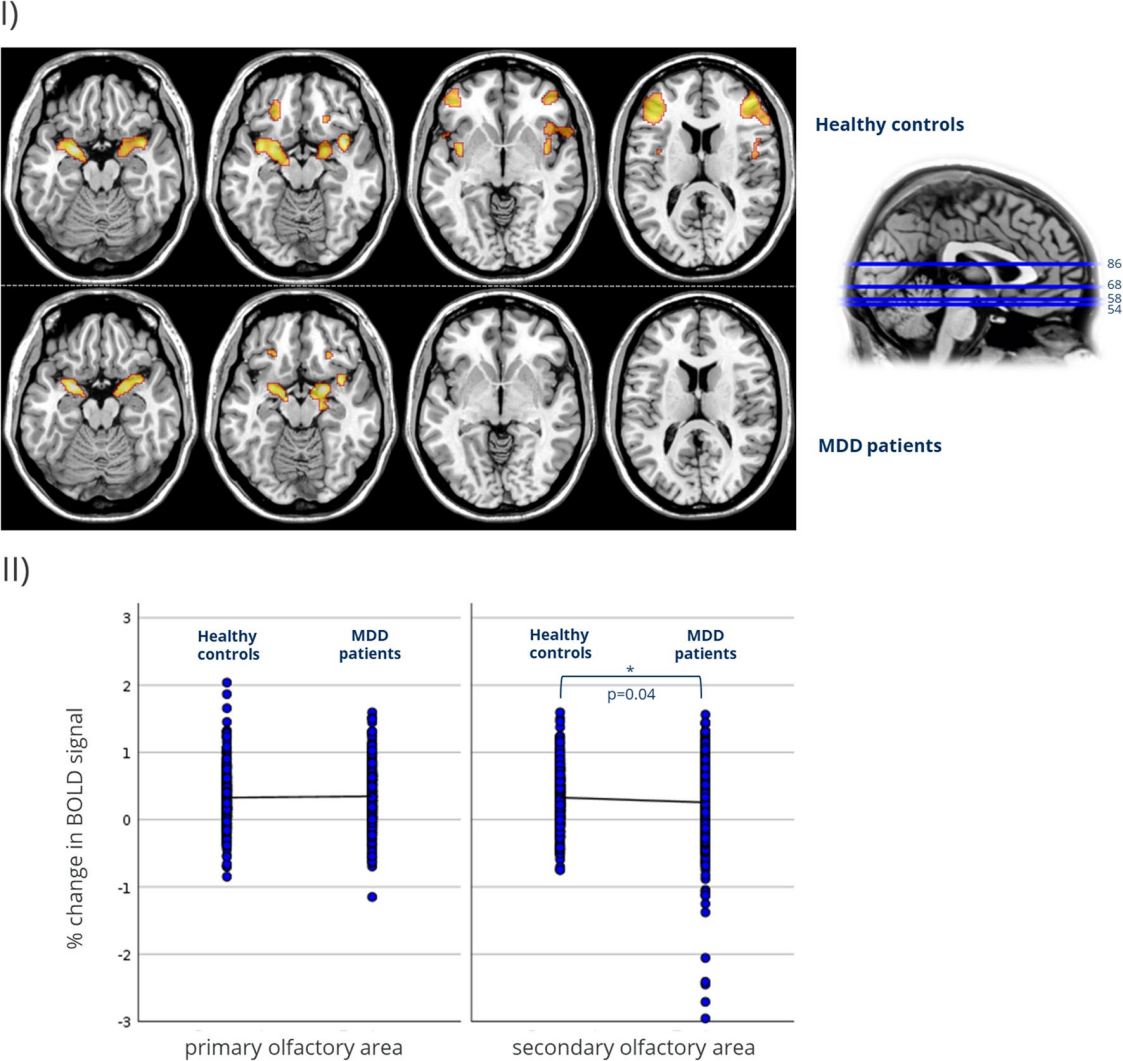
(I) Brain activation for the odors vs. baseline condition in healthy controls and depressed patients (MDD). Activation patterns for both groups are shown FWE-corrected (p < 0.05); axial planes. The location of the slice planes is indicated on the T1-weighted anatomical scan. Visualization follows the radiologic convention, hence structures visualized on the left side in the axial sectional image are located on the right side. (II) Percentage of BOLD signal change in primary and secondary olfactory areas in healthy controls and MDD patients. BOLD signal estimates of predefined olfactory ROI were extracted using Marsbar^45^. For analysis purposes piriform cortex, entorhinal cortex and amygdala were pooled as primary olfactory area, Hippocampus, Thalamus, Insula, Orbitofrontal Cortex were considered secondary olfactory areas. The Y-axis shows the percentage of BOLD signal change, with each horizontal line indicating a 1% change from the baseline activation within the respective olfactory ROI area. While BOLD activation levels in the primary olfactory areas did not differ between groups (T[2012] = − 0.31; p = 0.76), activation in the secondary olfactory areas was significantly lower in patients compared to controls (T[2012] = 2.1; p = 0.04).

Both groups exhibited significant increases in BOLD signal in almost all olfactory processing areas when odorous cues were presented. Following the olfactory ROI analysis, whole brain analysis revealed large additional activations, mostly within the frontal cortex, among healthy controls. Activations following pooled odor presentation are shown in Fig. 2 and Table 2.

**Table 2:** Effect of odor presentation in both groups.

		Healthy controls					MDD patients				
		MNI Coord.			k	T	MNI Coord.			k	T
		x	y	z			x	y	z		
Primary olfactory area											
Piriform Cortex	R	20	0	− 14	23	6.24	20	0	− 14	33	7.58
	L	− 18	0	− 16	19	6.14	− 18	0	− 16	19	6.14
Entorhinal Cortex	R	22	0	− 16	17	6.01	22	0	− 16	18	7.34
	L	− 24	2	− 18	53	8.18	− 24	2	− 18	45	7.67
Amygdala	R	20	− 2	− 16	23	6.27	20	− 2	− 16	33	7.15
	L	− 22	− 4	− 16	46	6.57	− 14	− 4	− 16	21	6.54
Secondary olfactory area											
Thalamus	R	8	− 2	4	34	5.77					
	L	− 12	− 4	8	9	4.87					
Anterior Insula	R	38	8	− 12	191	8.21	36	8	− 14	63	6.60
	R	− 38	4	− 10	127	8.13	44	18	− 6	3	4.67
	L	− 38	− 6	8	17	4.91	− 28	8	− 18	8	5.43
	L	− 34	22	0	18	4.91					
Posterior Insula	R	38	6	− 14	45	7.58	36	6	− 14	9	6.03
	L	− 38	2	− 10	40	7.79					
Hippocampus	R						22	− 14	− 14	7	5.68
	L	− 14	− 10	− 16	5	5.38					
Orbitofrontal Cortex (OFC)	R	22	30	− 14	7	5.09	26	30	− 14	8	5.16
	R	24	8	− 18	7	4.87	24	8	− 18	3	5.12
	R	46	50	− 10	6	4.80					
	R	38	26	− 10	2	4.66					
	L	− 24	36	− 12	67	6.66	− 26	8	− 22	6	5.71
	L	− 24	8	− 16	6	5.89	− 24	34	− 12	23	5.30
	L	− 42	52	− 10	15	5.04					
Whole brain analysis (k = 5)											
TrIFG (BA45)	R	42	44	2	175	6.65					
	L	− 44	42	6	263	6.44					
OrIFG (BA47)	R	44	24	− 8	38	5.14	48	22	− 8	4	4.83
OpIFG	R	52	14	4	71	5.22					
	R	56	26	12	12	5.18					
	L	− 52	12	0	12	4.90					
Middle frontal gyrus (MFG)	R	46	48	4	461	7.25					
	L	− 38	38	12	413	6.46					
Central operculum	R	46	− 4	14	10	4.78					
	L	− 38	0	16	5	4.78					
Frontal operculum	R	56	12	0	43	5.01					
	L	− 50	14	− 2	3	4.74					
Caudate	R	12	2	10	23	5.21					
Pallidum	L	− 22	− 8	− 8	6	5.15					

Group differences in activation	Healthy controls > MDD patients						MDD patients > Healthy controls				
		MNI Coord.			k	T	MNI Coord.			k	T
		x	y	z			x	y	z		
Primary olfactory area											
	No suprathreshold cluster						No suprathreshold cluster				
Secondary olfactory area											
Orbitofrontal cortex	L	− 42	54	− 8	10	3.44					
Whole brain analysis (k = 5)											
TrIFG	R	42	44	2	15	3.85					
Middle frontal gyrus	R	42	46	4	77	4.03					

Brain activation in olfactory and depression-related brain areas after odor stimulation. Chosen contrast is pooled odors vs clean air baseline (Odors ON vs OFF). Further, brain activation for pooled odors as contrast between groups is displayed. Where cells are empty, no suprathreshold cluster was observed.

*TrIFG* Triangular part of the Inferior frontal gyrus, *OrIFG* Orbital part of the Inferior Frontal Gyrus, *OrIFG* Opercular part of the Inferior Frontal Gyrus; *k* Cluster size in Voxel; *T* Peak T Value, p_FWE_ < 0.05 for effect of odor presentation per group, p < 0.001(uncorr.) for pooled odors as contrast between groups, *R* Right, *L* Left.

Comparing odor to baseline activation patterns, there was no significant effect of group (F[1,2012] = 1.1; p = 0.296), but a tendency for group by ROI interaction (F[2,2012] = 2.8; p = 0.063). While BOLD activation strength did not differ between groups in the primary olfactory areas (T[2012] = − 0.31;p = 0.76), there was a significantly lower activation in patients as compared to controls in the secondary olfactory areas (T[2012] = 2.1; p = 0.04). Comparisons between patients and healthy controls for BOLD activation within primary and secondary olfactory areas for the overall effect of the odor stimulation revealed a significantly reduced activation of the left orbitofrontal cortex (− 42 54 − 8) in patients. For the same contrast the exploratory whole brain analysis indicated significantly less activation of the right inferior frontal gyrus (42 44 2) and middle frontal gyrus (42 46 4; − 42 56 − 6) in the patient group.

## Discussion

The association between olfactory abnormalities and depression is complex. Despite decades of research the underlying mechanisms remain poorly understood. Using structural and functional MRI as well as psychophysical tests, we re-evaluated theoretical assumptions and previous findings about the relationship between depression and olfactory functions. Based on careful selection of participants, piloting of odors and use of already established olfactory tests and imaging procedures, results of earlier studies were partially replicated.

Whereas it should be noted that studies are at odds as to which dimension of olfactory perception is altered in depression, they overall agree that at least portions of the sense of smell are often affected in MDD patients^3^. Frequently reported are reduced odor discrimination, odor threshold and hedonic evaluations of odors which is consistent with our present findings. Patients experienced significant difficulties in correctly recognizing and discriminating the odors presented in the fMRI runs. In contrast, none of the 21 healthy control subjects had such difficulties. Further, our analysis revealed a reduced odor sensitivity in MDD patients as measured with the Sniffin’ Sticks threshold test. The effect size was relatively high (Cohen’s d = 0.49). It is reasonable to expect the group comparison of olfactory sensitivity to become statistically significant with a larger number of subjects. Our results do not indicate a group difference with regard to odor identification, which is in line with previous findings^11,26,46–48^. In overview, a reduction in the olfactory ability in people with depression is often described in the literature, but this is not as pronounced in our findings. Rather, our data, as well as others, show that some of the depressed patients also have an unimpaired ability to smell. This suggests that there are different courses of odor impairment in depression or different subcategories. For instance, olfactory impairment is more pronounced in recurrent depression than in single episode and in depression with longer duration^49^. In terms of hedonic ratings of the odors presented during the fMRI scans, ratings of MDD patients were more oriented towards neutral compared to healthy controls. We assume that the less pronounced hedonic evaluation is linked to the severity of depressive symptoms, since the BDI and hedonic ratings showed a significant negative correlation. Patients who scored high on the BDI were also found to have high levels of anhedonia as determined by the SHAPS questionnaire. The direct relationship between olfactory anhedonia and a patient’s clinical anhedonia has already been demonstrated^10^. The blunted hedonic ratings of the odors may therefore be considered as an expression of anhedonia, a core symptom of depression characterized by a diminished ability to experience pleasure^50^.

Next, we conducted structural MRI to examine the OB volume for both groups. The absence of OB has been linked to increased susceptibility to depression in both rodents and humans^25,51^. Some studies have also shown a correlation between the size of the OB and depressive symptoms in MDD patients^26,27^. In this context, it has also been suggested that reduced input from a small OB reaching amygdala and hippocampus may compromise adequate emotional performance. Hence, a smaller OB could be related to the development of depression via its connections to limbic areas. Contrary to previous studies^26,27^, in terms of OB volume we found no significant group difference. As size and volume of the OB is thought to be generally stable during antidepressant therapy^52^, our finding is not attributable to a change linked to the course and treatment of depression. We discuss our result considering the improved imaging options. In previous studies, surface measurements of the individual layers were added and multiplied by a factor corresponding to the layer thickness to obtain an estimated total volume. We decided to use a continuous interslice-gap free acquisition of brain images to optimize precise measurement instead of relying on estimated volumes. In addition, we also performed MRI with a much higher image resolution, choosing an ISO voxel size of 0.5 × 0.5 × 0.5 mm instead of 2 × 2 × 2 mm, which most likely also yields a more precise measurement of anatomical structures. Based on our result, we cannot confirm previously described OB reductions in MDD patients. A preceding study was also unable to find a significant reduction in OB volume in treatment-responsive depressed patients both before and after therapy compared to healthy controls^52^. Moreover, we could not find a correlation between OB volume and averaged BOLD signals in any downstream brain areas of olfactory and emotional relevance including the amygdala, the hippocampus and OFC. A direct relationship between OB volume and limbic circuit modulation, as suggested and considered originally for the purpose of this study, is thus not supported by our findings.

Functional MRI results revealed a solid activation of almost all primary and secondary olfactory areas following odor presentation, with similar activation patterns in both groups. Olfactory stimulation resulted in significant and strong BOLD responses in the olfactory brain areas, consistent with previous studies about central odor processing^53^ and thus supporting the validity of the present study design. There was no difference in the perceived intensity of the presented odors between groups, group differences in fMRI activation can thus not be attributed to differences in intensity of the odors. While BOLD activation strength did not differ between groups in the primary olfactory areas, our analysis revealed a significantly lower activation in the secondary olfactory areas in patients as compared to controls. Further comparisons between groups provided more specific insight into brain area activation trends but were conducted by lowering the global height threshold. Please keep in mind, that the statistical thresholds of p < 0.001 is very liberal. While this allows detection of smaller effects, it also enhances the risk for false positive results. Hence, a replication of this study is especially warranted. The threshold setting is consistent with the only currently existing comparable study focusing on central olfactory processing in depression^11^. In line with our results, this study reported a reduced activation of secondary olfactory areas comparing healthy controls and MDD patients. However, whereas our analysis indicates an overall difference in the activation of secondary olfactory areas, most notably in the OFC, the previous study also reported small clusters of decreased BOLD activation in the insula and thalamus in addition to the OFC. Before we attempt to frame our results, it is worth considering our findings in the context of the limitations of the previous study. While the sample of patients had a similar severity of depressive symptoms compared to our patient group, only women with a history of severe childhood maltreatment and post-traumatic stress disorder were investigated. The sample is therefore unlikely to be representative for all depressed patients. As depression is often accompanied by other psychiatric disorders, subsidiary diagnoses could also not be completely avoided in our sample. However, experiences of threat and trauma are particularly drastic events that may bias the results of the previous study. In addition, there is also a difference between the two studies in terms of image acquisition. Instead of performing the fMRI scans with a 1.5 T scanner, results from the present study were based on advanced 3 T techniques. This presumably also increased the likelihood of capturing small effects. Hence, there were a few but significant technical differences between the two studies. However, both studies agree that there seem to be no major differences between controls and MDD patients in the very basic processing of odorous cues as primary olfactory areas show similar activation patterns. Instead, results imply group differences in the processing of odors to be mainly attributable to secondary olfactory areas and higher order processing areas. A reduced activation in the OFC of patients with depression was observed in both studies. The OFC is involved in complex information processing including value attribution to olfactory stimuli^15^, allocation of attentional resources^16^, olfactory awareness and conscious perception^54^ and evaluative odor judgements^18^. Its activation is also regularly accompanied by regional co-responses in large parts of the frontal cortex, suggesting the engagement of non-olfactory networks in higher-level odor judgements^9^. This network of intracerebral connections between olfactory regions and other brain areas is believed to enable the refined integration of olfactory clues with memories and emotions^55^. Further, a recent meta-analysis concluded that the OFC and other frontal brain areas such as the middle and inferior frontal gyrus are linked to conscious smelling with odor evaluations rather than passive smelling^56^. Accordingly, we observed extensive activation of frontal brain areas in addition to standard olfactory areas in the group of healthy subjects in our broader whole brain analysis. Our participants were likewise instructed to consciously perceive and subsequently rate the odors. However, in the depressed group, we found significantly less activation of both the OFC and surrounding prefrontal areas. As mentioned in the introduction, decreased metabolic activity in frontal cortical regions is generally a common observation in studies investigating depression^19,57^. With the background of positive regulatory downstream effects of TMS on limbic circuits^20^ and improvement of olfactory function^21^, we tried to frame the reduced activation of secondary olfactory areas with emphasis on the OFC observed in our patient group. Abnormalities in the frontal cortex are believed to lead to secondary impairments in subcortical areas and trigger depression in general^19^. We are speculating, that reduced activation of frontal brain areas of higher cognitive order might limit feedback to limbic structures leading to problems in emotion regulation and hedonic value attribution to odors. This may interfere with the conscious perception of odors and thus contribute to olfactory impairments in depression. A dysfunction in these brain areas has already been suggested to explain deficits in hedonic rating in depression due to its integrative function^10^. This can be aligned with the observed psychophysical results and odor ratings. Hedonic perception of odors is among the major characteristics of olfactory perception, mainly shaped by previous experience^58^. Inadequate engagement of frontal brain areas may disturb necessary integration of basic sensory input and contextual memory associations during olfaction in depression, eventually causing blunted hedonic ratings as observed in the patient group. Consistent with previous studies, we also observed reduced odor discrimination ability in the patient group^11,59^. This subtype of olfactory testing is thought to reflect executive functions^4^, suggesting abnormalities in higher order processing in depressed patients. A relationship between difficulties in higher order olfactory performance and OFC function has already been described in individuals with an OFC lesion, showing remarkable difficulties in odor discrimination and memory^60^. Reduced activity in the OFC as observed in the group of patients seems to have a similar effect. In contrast, increased fMRI signals in the OFC were associated with an improvement in the ability to discriminate odors in healthy individuals^61^. Discrimination of successively presented odors requires working memory to briefly store the preceding odor’s perceptual trace to be subsequently compared to the following odors in the group as well as adequate attribution of attention to the properties of the odor. MDD patients often complain about attention difficulties that negatively affect many aspects of daily functioning^62^. We assume that a limitation of attention, reflected in reduced activation of secondary olfactory areas and regions of higher order processing in frontal brain areas, might be an important driver for olfactory impairment in depressed patients. Besides discrimination and hedonic judgements, attention can influence all domains of olfactory perception. It has previously been suggested that attentional impairments in depression may even have a negative effect on the turn-over rate at the olfactory epithelium^3^. This in turn could explain the worsened threshold in patients, which has been observed as a tendency in the present patient group but also in previous research^26^. A gradual improvement of the odor threshold after successful drug medication^63,64^ might be explained by a drug-related increase of attention, boosting the turnover rates at the olfactory epithelium and subsequently promoting improvement of olfactory ability^3^.

There are a few limitations to this study. Depression is often coexistent with other psychiatric disorders, therefore secondary diagnoses could not be completely avoided in our sample of patients. Eating disorders in particular, however, were an exclusion criterion as olfactory dysfunction related to abnormal eating behaviour has previously been reported^65^. Another potential explanation for differences in odor testing performance and BOLD signal activation may also lie in the different medication preferences within the patient group (drug, dosage, exclusively psychological therapy). In the present study, a limited number of study participants didn’t allow for a meaningful subgroup analysis. Future studies may tackle this point by excluding patients with medication.

Sniffing could be a confounding factor in olfactory fMRI^66^. Prior to the fMRI scans, all participants were therefore instructed to avoid sniffing and only breathe passively through the nose. However, as sniffing patterns were not controlled for, a possible effect cannot be ruled out. Further, if a breathing belt is available, the breathing pattern should be recorded for each participant during the fMRI experiments so that it can later be accounted for in the analysis from the fMRI data.

## Conclusion

Our findings suggest no major differences between controls and MDD patients in the very basic processing of odorous cues as primary olfactory areas show similar activation patterns.

Instead, the present results imply that group differences in the processing of odors may mainly be attributable to secondary olfactory areas and higher order processing areas within the frontal cortex. The OFC seems to be most affected by this, however this observation must be taken with caution. Considering the numerous functions of the OFC including value attribution, olfactory awareness, and cognitive higher-order processing, we are in favor of a top-down mechanism explaining, at least in part, the relation between depression and olfaction. We suggest that reduced activation of secondary olfactory areas and the frontal cortex in MDD patients provide reason that integrative and attentional resources may be inadequately allocated to the odors. This could be a mechanism for impaired hedonic evaluations and olfactory discrimination in patients. Reduced activity in cortical areas of higher order processing and secondary impairment of olfaction may constitute an integral part of the relationship between depression and olfaction.

## Supplementary Information

Supplementary Information.

## Data Availability

The dataset used in this article is available upon a formal and reasonable request from the corresponding author.
