# Supplementary material for: Olfactory brain activations in patients with Major Depressive Disorder
Source: Sci Rep. 2023 Jun 21;13:10072. doi: 10.1038/s41598-023-36783-0 (PMC10284830; doi:10.1038/s41598-023-36783-0)
Supplement: Supplementary file 1 — Supplementary Information. [file 41598_2023_36783_MOESM1_ESM.docx]

**Supplementary information**

There was no significant difference of the intensity of odors with Lilac and Chocolate kept at neat concentration and tar in 10%dilution, ANOVA: F(2,30)=0.90, p=0.417.

**
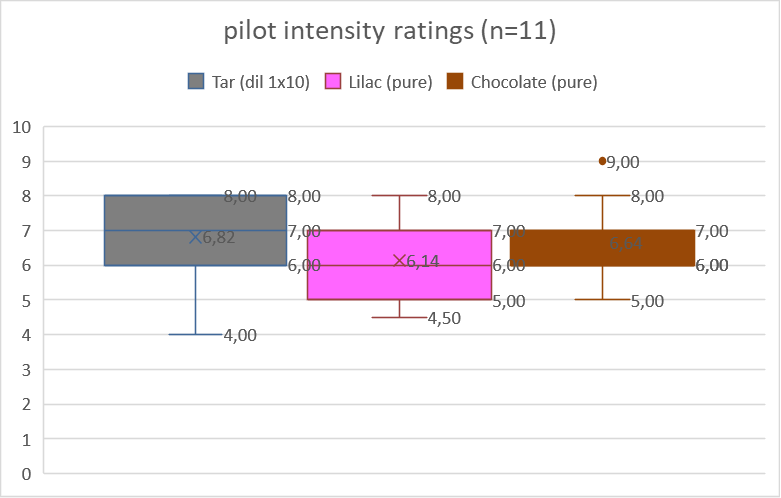

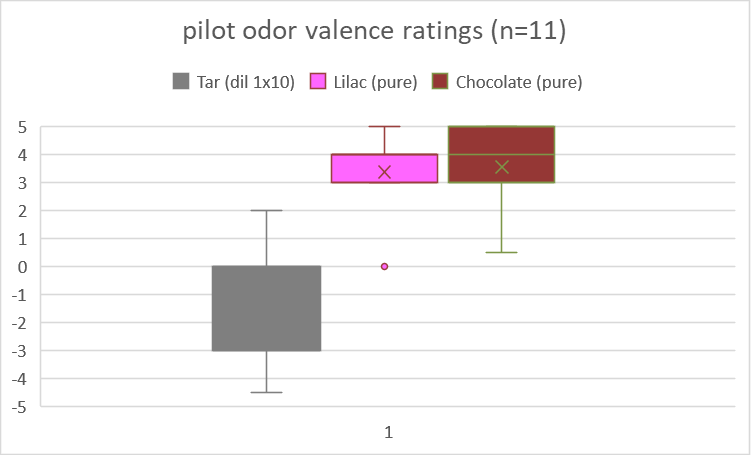
**

**Subgroup analysis grouped by Odor Discrimination and color-coding of the subgroups in the plots**

This was a valuable propose by one of our reviewers. The graph does not give the impression that a reduced ability to discriminate odors (score 7 and 8) is the driver of olfactory differences between healthy controls and MDD patients.

Regrettably, a group size of 21 patients is too small for meaningful subgroups analyses to be performed.
Nevertheless, out of own curiosity we have modelled an identical GLM with a subgroup of patients (n=12) that had no difficulties in discrimination. Interestingly, however, this reinforced the impression of different olfactory processing between the groups. Comparing odor to baseline activation patterns, there was a significant effect of group (F[1,1628]=13.3; p<0.001), accompanied by an identical tendency for group by ROI interaction (F[2,1628]=2.8; p=0.063). While BOLD activation strength again slightly missed significancy between groups in the primary olfactory areas (T[2012]=1.9;p=0.06), there was a significantly lower activation in patients as compared to controls in the secondary olfactory areas (T[2012]=4.0; p<0.001).
